# Supplementary material for: Comparative analysis of nuclei isolation methods for brain single-nucleus RNA sequencing
Source: Cell Rep Methods. 2026 Mar 23;6(3):101337. doi: 10.1016/j.crmeth.2026.101337 (PMC13030979; doi:10.1016/j.crmeth.2026.101337)
Supplement: Document S1. Figures S1 and S2 [file mmc1.pdf]

Cell Reports Methods, Volume *6*

Supplemental information

Comparative analysis of nuclei isolation methods for brain single-nucleus RNA sequencing

Holly N. Kersey, Dominic J. Acri, Luke C. Dabin, Kelly A. Hartigan, Richard Mustaklem, Jung Hyun Park, and Jungsu Kim

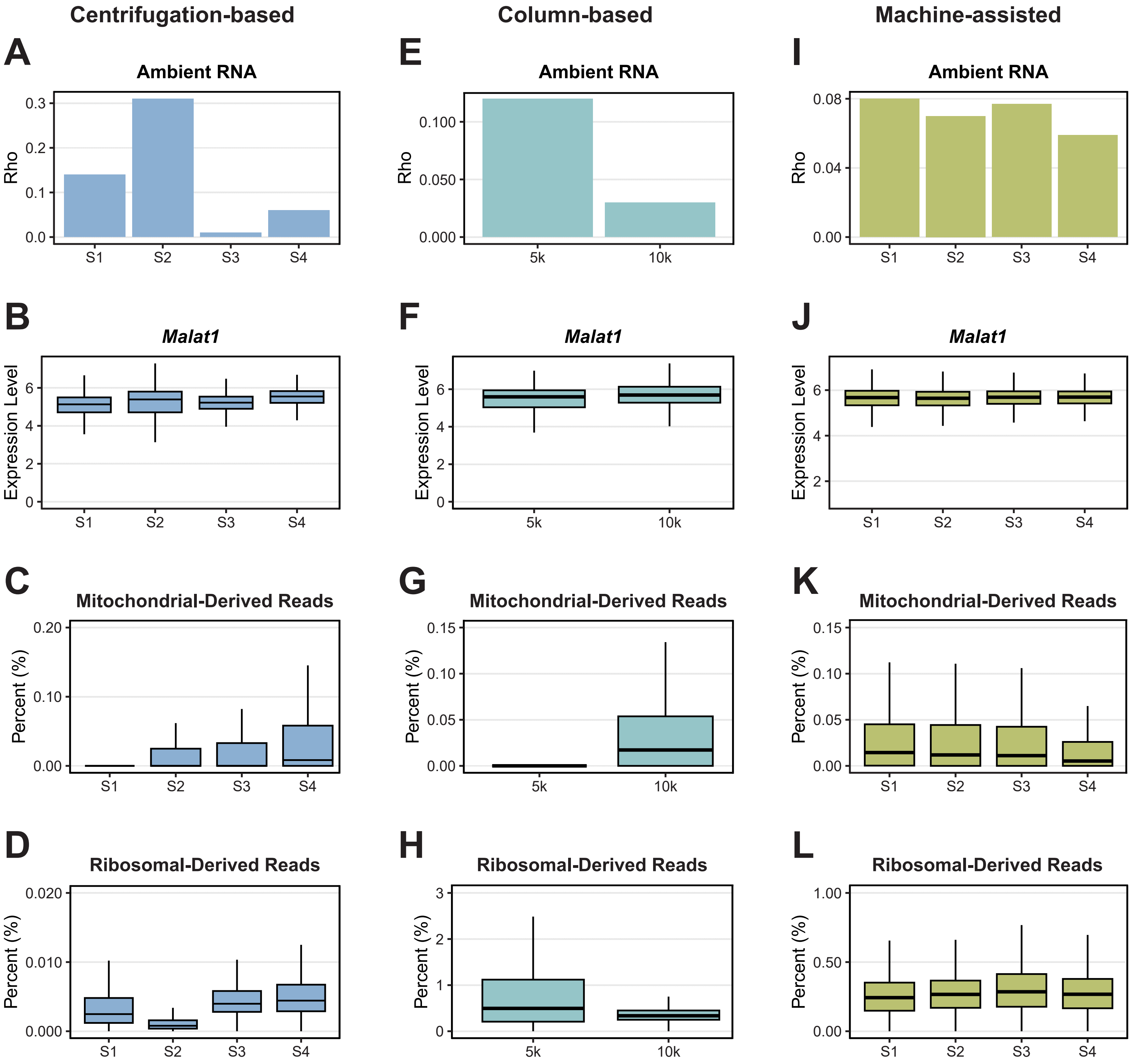

Supplemental Figure 1: Quality control metrics from additional experiments, related to Figure 3. Additional datasets from each nuclei isolation method were analyzed: four samples (S1-S4) with the centrifugation-based method (**A**-**D**), two publicly available datasets from 10X Genomics [5k (5k Adult Mouse Brain Nuclei Isolated with Chromium Nuclei Isolation Kit), 10k (10k Adult Mouse Brain Nuclei Isolated with Chromium Nuclei Isolation Kit, Chromium NextGEM Single Cell 3')] for the column-based method (**E**-**H**), and four samples (S1-S4) with the machine-assisted method (**I**-**L**). Samples were analyzed for ambient RNA (**A**,**E**, and **I**), *Malat1* expression (**B**, **F**, and **J**), mitochondrial-derived reads (**C**, **G**, and **K**), and ribosomal-derived reads (**D**, **H**, and **L**). Boxplots display distribution of data where middle line represents the median. The edges of the box indicate the first and third quartiles. The whiskers extend to the minimum and maximum values within 1.5 times the inter-quartile range from the quartiles.

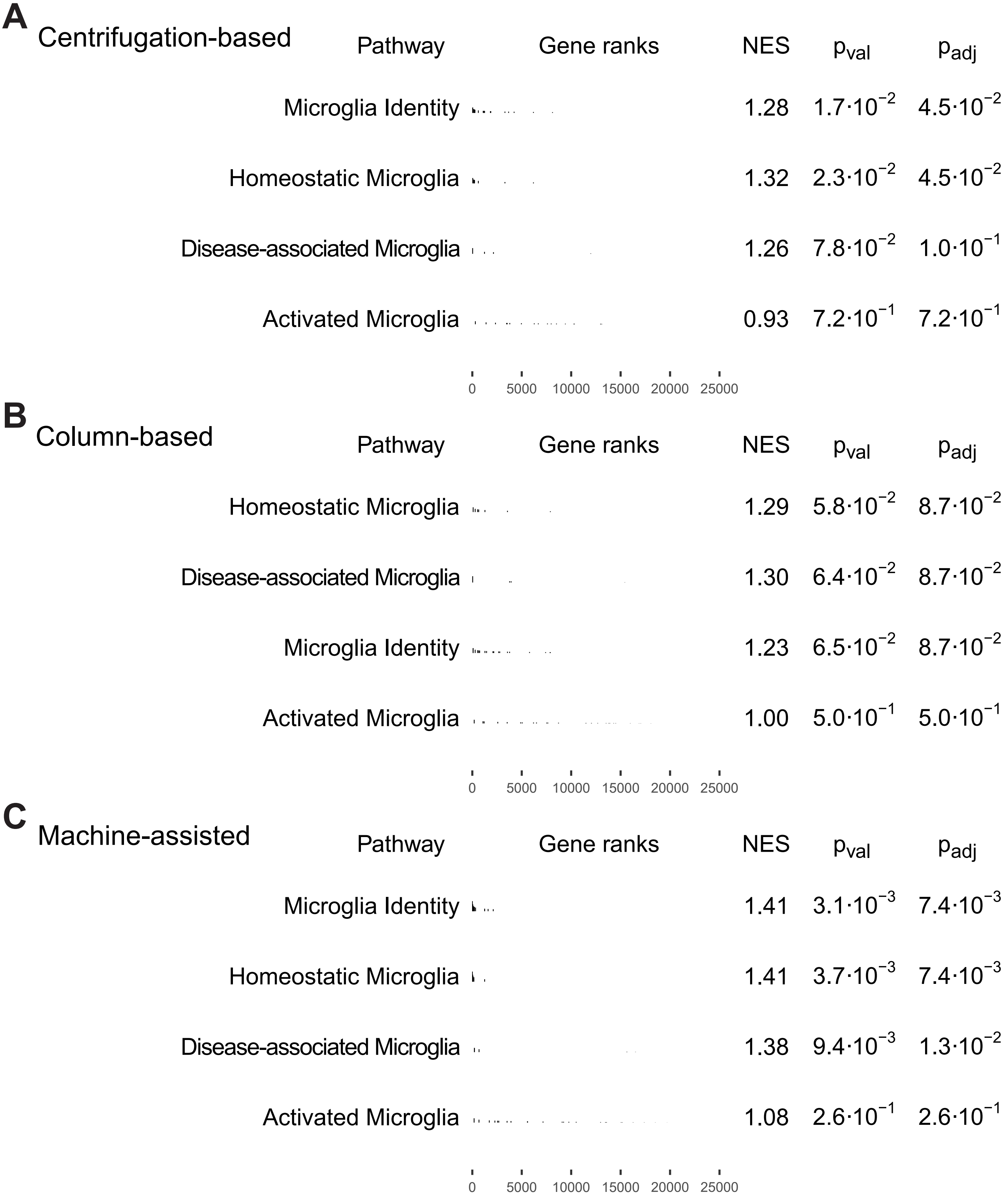

Supplemental Figure 2: Gene set enrichment analysis (GSEA) for microglial *ex vivo* activation signature, related to Figure 4.

GSEA results comparing four microglia gene signatures in the (**A**) centrifugation-based method, (**B**) column-based method, and (**C**) machine-assisted method. "Microglia Identity" and "Activated Microglia" (*ex vivo* microglial activation) signatures from Marsh et al. (2022). For each pathway, the Gene ranks column displays the distribution of genes within each signature across the ranked gene list (lines represent individual genes) with the Normalized Enrichment Score (NES), nominal p-value (pval), and false discovery rate-adjusted p-value (padj). Positive NES values indicate enrichment at the top of the ranked gene list.
